# The Strengths and Difficulties Questionnaire as a Valuable Screening Tool for Identifying Core Symptoms and Behavioural and Emotional Problems in Children with Neuropsychiatric Disorders

**DOI:** 10.3390/ijerph19137731

**Published:** 2022-06-23

**Authors:** Melissa Grasso, Giulia Lazzaro, Francesco Demaria, Deny Menghini, Stefano Vicari

**Affiliations:** 1 Neurological and Neurosurgical Diseases Research Unit, Bambino Gesù Children’s Hospital IRCCS, 00146 Rome, Italy; melissa.grasso@opbg.net; 2 Child and Adolescent Neuropsychiatry Unit, Department of Neurosciences, Bambino Gesù Children’s Hospital IRCCS, 00146 Rome, Italy; giulia.lazzaro@opbg.net (G.L.); francesco.demaria@opbg.net (F.D.); stefano.vicari@opbg.net (S.V.); 3 Department of Life Sciences and Public Health, Catholic University, 00168 Rome, Italy

**Keywords:** transdiagnostic, mental health, assessment, psychopathology, screening

## Abstract

The Strengths and Difficulties Questionnaire (SDQ) is a worldwide questionnaire used for the early identification of behavioural/emotional symptoms in children and adolescents with neuropsychiatric disorders. Although its prognostic power has been studied, it has not yet been tested whether SDQ: (i) can identify pathognomonic symptoms across a variety of neurodevelopmental and neuropsychiatric disorders, (ii) can capture emotional and behavioural problems associated with the main diagnosis, as well as shared transdiagnostic dimensions, and (iii) can detect changes in symptomatology with age. The present study evaluated nearly 1000 children and adolescents overall with Global Developmental Delay (GDD), Intellectual Disability (ID), Language Disorder (LD), Specific Learning Disorder (SLD), Autism Spectrum Disorder (ASD), Attention Deficit/Hyperactivity Disorder (ADHD), Mood Disorder (MD), Anxiety Disorder (AD), and Eating Disorders (ED). We found that SDQ: (i) can identify the core symptoms in children with ASD, ADHD, MD, and AD via specific subscales; (ii) can capture the associated emotional and behavioural symptoms in children with LD, GDD, ID, SLD, and ED; and (iii) can detect changes in the symptomatology, especially for GDD, LD, ASD, ADHD, and AD. SDQ is also able to recognise the transdiagnostic dimensions across disorders. Our results underscore the potential of SDQ to specifically differentiate and identify behavioural/emotional profiles associated with clinical diagnosis.

## 1. Introduction

Childhood and adolescence are sensitive periods characterised by determinant changes in the physical, emotional, and social domains [1,2]. During these periods, individuals are often exposed to high levels of psychological distress that may compromise their psychophysics well-being and make them more vulnerable to the development of neuropsychiatric disorders [3]. Neuropsychiatric disorders are often described as the leading cause of disability in youths worldwide, with significant costs to families, individuals, and national health systems [4] and harmful consequences during development.

Globally, an estimated 10–20% of youths usually experience mental health conditions that often remain underdiagnosed and undertreated [3], with detrimental consequences during development. An example is Attention Deficit/Hyperactivity Disorder (ADHD), one of the most common and disabling neurodevelopmental disorders of childhood. When misdiagnosed and never treated, ADHD persists into adulthood in association with aggravating comorbidities, including mood disorders (MD) and anxiety disorders (AD), conduct disorder, antisocial personality and substance abuse, bipolar disorder, impulse control disorders, and even suicidal behaviour [5,6,7,8,9]. Similarly, if left unrecognised and untreated during the developmental ages, neuropsychiatric disorders such as MD and AD are likely to be steady or even to become more acute from adolescence to midlife, becoming increasingly difficult to treat as time goes on [10,11,12].

Of importance, most neurodevelopmental disorders, including Autism Spectrum Disorders (ASD) and Intellectual Disability (ID), as well as Specific Learning Disorders (SLD) and Language Disorders (LD), are commonly associated with poorly recognised behavioural and emotional problems [13,14,15,16,17,18,19,20] and not infrequently result in psychiatric disorders.

Early screening offers an important opportunity to rapidly detect and treat behavioural and emotional symptoms. Therefore, it is not surprising that, over the past two decades, several studies [21,22,23,24,25] have attempted to identify the early neuropsychiatric symptoms in children and adolescents via psychological questionnaires.

One worldwide screening tool used to detect neuropsychiatric symptoms in children and adolescents is the Strengths and Difficulties Questionnaire (SDQ) [26]. With good psychometric properties [23,27,28], the SDQ is a brief flexible questionnaire used for the early screening and identification of pathognomonic symptoms or behavioural and emotional symptoms associated with neuropsychiatric disorders, which require further evaluation with a structured neuropsychiatric examination. The SDQ has been employed in several studies to screen for neuropsychiatric disorders, including ADHD [29,30] and ASD [31], or to identify emotional and behavioural problems in children and adolescents with neurodevelopmental disorders [21,22,25,32], such as SLD [33] and ID [34].

Furthermore, a recent longitudinal study [35] demonstrated the predictive validity of the SDQ in a population of 1176 children. Specifically, pre-schoolers with the highest scores on the SDQ were at a higher risk of having symptoms of ADHD, behavioural disorders (oppositional defiant disorder or conduct disorder), and emotional disorders (depression or AD) in preadolescence.

A recent study by Vugteveen et al. [36] investigated to what extent specific SDQ profiles were associated with neuropsychiatric diagnosis based on the Diagnostic and Statistical Manual of Mental Disorders-Fourth Edition (DSM-IV) criteria [37] among diagnosed adolescents. The results showed that SDQ identified pathognomonic symptoms that matched the DSM-IV diagnoses for almost 90% of adolescents (i.e., ADHD, conduct disorder/oppositional defiant disorder, ASD, MD, and AD). Moreover, SDQ recognised additional emotional and behavioural problems that were comorbid with the core diagnoses and were shared transdiagnostically. However, the study considered only a selected range of age (12–17 years) and disregarded several neurodevelopmental disorders, such as SLD, ID, and LD, and neuropsychiatric disorders, such as Eating Disorders (ED) [36].

The present study aimed to investigate whether the SDQ is a reliable and valid tool for identifying the relevant symptoms in nearly one thousand children and adolescents diagnosed with a variety of neurodevelopmental and neuropsychiatric disorders. The present study also aimed to explore whether the SDQ could be considered a screening tool for the recognition of shared transdiagnostic dimensions in terms of the behavioural and emotional problems associated with neurodevelopmental and neuropsychiatric disorders. Finally, the present study aimed to test whether the SDQ is a sensitive measure to capture changes in symptomatology with age.

If the SDQ were a reliable and valid screening questionnaire, we would expect that it could detect symptoms of neuropsychiatric disorders (e.g., hyperactive symptoms for ADHD; emotional problems for MD and AD; and peer and prosocial problems for ASD), as well as associated behavioural and emotional problems (e.g., conduct disorders for ADHD, emotional problems for ADHD and ED, and peer and prosocial problems for ID).

## 2. Materials and Methods

### 2.1. Participants and Procedures

Out of 1000 children and adolescents attending the Child and Adolescent Neuropsychiatry Unit of the Bambino Gesù Children’s Hospital for an initial screening neuropsychiatric visit, a group of 952 participants were further referred for an in-depth neuropsychiatric examination and selected between December 2016 and March 2019 (Males, M/Females, F: 606/346; age range: 0.7–19.1 years; mean age ± standard deviation (SD): 8.9 ± 4.8 years).

Participants underwent a child neuropsychiatric examination conducted by experienced developmental neuropsychiatrists. The clinical diagnosis derived from the developmental history and the clinical examination based on the Diagnostic and Statistical Manual of Mental Disorders, 5th Edition (DSM-5) criteria [38].

Participants were divided into nine groups based on the clinical diagnosis according to the DSM-5 criteria [38] as follows: 113 participants showed Global Developmental Delay (GDD; M/F: 89/24; 4.2 ± 1.8 years), 45 participants had ID (M/F: 27/18; 12.4 ± 4.5 years), 172 LD (M/F: 122/50; 4.3 ± 1.6 years), 187 SLD (M/F: 96/91; 12 ± 3 years), 69 ASD (M/F: 54/15; 7.3 ± 4.4. years), 131 ADHD (M/F: 110/21; 8.5 ± 3.7 years), 51 MD (M/F: 20/31; 13.7 ± 3.6 years), 151 AD, including obsessive-compulsive disorder and tic disorder (M/F: 82/69; 12 ± 4.1 years), and 33 ED (M/F: 6/27; 14 ± 3.7 years).

### 2.2. Measures

#### Strengths and Difficulties Questionnaire

SDQ [39] is a brief 25-item behavioural screening questionnaire for children and adolescents asking the parent to what extent both the positive and negative psychological attributes of the child were true in the past six months using a 3-point Likert scale (0 = not true, 1 = somewhat true, and 2 = certainly true). The SDQ consists of five subscales, each consisting of five items: Emotional Problems, Conduct Problems, Hyperactivity/Inattention, Peer Problems, and Prosocial Behaviour. In the study, we used raw SDQ scores, according to the original three-band categorisation (normal, borderline, and clinical) [39]. We chose the parent form of SDQ, because it would seem that the parents, as informants, are more cautious in describing their children’s problems compared to the self-reports of youngers [40].

The SDQ has been validated for use in many countries across different languages, and it is commonly adopted in many European and Extra-European countries, including the United Kingdom [27], Finland [40], Germany [41], Sweden [42], and Italy [43,44]. All published studies confirmed the good psychometric properties of the instrument and the excellent balance between the time taken to fill it in and the amount of information collected. The SDQ can be obtained without charge from a website (https://www.sdqinfo.com) (accessed on 1 December 2016), and therefore, it is practical and free to use within clinical settings.

### 2.3. Statistical Analysis

The percentage of participants who obtained normal, borderline, and clinical scores in each SDQ subscale was calculated.

Diagnostic groups (GDD vs. ID vs. LD vs. SLD vs. ASD vs. ADHD vs. MD vs. AD vs. ED) were compared on the SDQ subscales (Emotional Problems vs. Conduct Problems vs. Hyperactivity/Inattention vs. Peer Problems vs. and Prosocial Behaviour) using the Kruskal–Wallis ANOVA, because the assumption of normality was not fulfilled. Bonferroni’s correction for multiple comparisons was applied to account for the five comparisons of diagnostic groups on each SDQ subscale, and a *p*-value ≤ 0.01 was considered significant (*p*-value = 0.05/5 SDQ subscales = 0.01). Post hoc comparisons across the diagnostic groups on each SDQ subscale were run by using the mean ranks of all pairs of groups [45].

To test whether the scores on the SDQ subscales were age-dependent, Spearman’s correlations (*rho*) between age and the scores on the SDQ subscales were run separately for each diagnostic group. Bonferroni’s correction for multiple comparisons was applied to account for the nine comparisons of the diagnostic groups within each SDQ subscale, and a *p*-value ≤ 0.0055 was considered significant (*p*-value = 0.05/9 and the diagnostic groups = 0.0055).

## 3. Results

Table 1 shows the percentage of children and adolescents who showed normal, borderline, and clinical scores on the SDQ subscales in each Diagnostic Group, as well as the mean and standard deviations (SD) of the scores in the SDQ subscales and post hoc comparisons.

The Diagnostic Groups differed in each SDQ subscale analysed: the Emotional Problems Subscale (H(8) = 166.15, *p* < 0.001), Conduct Problem Subscale (H(8) = 87.39, *p* < 0.0001), Hyperactivity/Inattention Subscale (H(8) = 144.11; *p* < 0.001), Peer Problems Subscale (H(8) = 110.47; *p* < 0.001), and Prosocial Behaviour Subscale (H(8) = 132.47; *p* < 0.001).

### 3.1. Global Developmental Delay

With the exception of the Emotional Problems Subscale, at least half of the participants with GDD showed a prevalence of clinical/borderline scores in the remaining SDQ subscales.

Comparisons between groups revealed that participants with GDD showed significantly worse scores in the Hyperactivity/Inattention Subscale than the groups with LD (*p* < 0.0001), AD (*p* < 0.001), and ED (*p* < 0.0001) but not compared to ID (*p* = 0.99), ASD (*p* = 0.99), MD (*p* = 0.99), SLD (*p* = 0.03), and ADHD (*p* = 0.04) groups.

In the Peer Problems Subscale, they also obtained worse scores than the SLD group (*p* = 0.009) and better scores than groups with ASD (*p* < 0.0001) but did not significantly differ from ID (*p* = 0.90), LD (*p* = 0.03), ADHD (*p* = 0.99), MD (*p* = 0.99), AD (*p* = 0.99), and ED (*p* = 0.99) groups.

Further, they obtained significantly worse scores in the Prosocial Behaviour Subscale than the groups with LD (*p* < 0.0001), SLD (*p* < 0.0001), MD (*p* = 0.005), AD (*p* < 0.0001), and ED (*p* < 0.001) but not than with ID (*p* = 0.95), ASD (*p* = 0.99), and ADHD (*p* = 0.02).

Conversely, they obtained significantly better scores in the Emotional Problems Subscale than the groups with MD (*p* < 0.0001), AD (*p* < 0.0001), and ED (*p* < 0.001) but not than ID (*p* = 0.05), LD (*p* = 0.40), SLD (*p* = 0.04), ASD (*p* = 0.99), and ADHD (*p* = 0.48) groups.

In addition, they obtained significantly better scores in the Conduct Problems Subscale only compared to the ADHD group (*p* < 0.0001) but did not differ from the ID, LD, SLD ASD, MD, AD, and ED (*p* always = 0.99) groups.

### 3.2. Intellectual Disability

With the exception of the Prosocial Behaviour Subscale, the majority of the participants with ID showed clinical or borderline scores in the Emotional Problems, Conduct Problems, Hyperactivity/Inattention, and Peer Problem Subscales. Of note, the highest percentage of clinical scores was in the Peer Problems Subscale (60%).

A comparison between the groups revealed that participants with ID exhibited significantly worse scores in the Emotional Problems Subscale than the groups with only LD (*p* < 0.0001) but not compared to GDD (*p* = 0.05), SLD (*p* = 0.99), ASD (*p* = 0.99), ADHD (*p* = 0.99), AD (*p* = 0.99), ED (*p* = 0.99), and MD (*p* = 0.02) groups.

Similarly, in the Hyperactivity/Inattention Subscale, they exhibited significantly worse scores than the groups with only ED (*p* = 0.002) but did not differ from the groups with GDD (*p* = 0.99), LD (*p* = 0.12), SLD (*p* = 0.99), ASD (*p* = 0.99), ADHD (*p* = 0.08), MD (*p* = 0.99), and AD (*p* = 0.37).

In the Peer Problems Subscale, they obtained significantly worse scores than the groups with LD (*p* < 0.0001) and SLD (*p* < 0.0001) but did not differ from the groups with AD (*p* = 0.01), GDD (*p* = 0.90), ASD (*p* = 0.99), ADHD (*p* = 0.20), MD (*p* = 0.99), and ED (*p* = 0.45).

Conversely, they obtained significantly better scores in the Prosocial Behaviour Subscale than the groups with only ASD (*p* = 0.006) but did not differ from the SLD (*p* = 0.04), GDD (*p* = 0.95), LD (*p* = 0.99), ADHD (*p* = 0.99), MD (*p* = 0.99), AD (*p* = 0.80), and ED (*p* = 0.90) groups, while, in the Conduct Problems Subscale, their scores did not significantly differ from the GDD (*p* = 0.99), LD (*p* = 0.21), SLD (*p* = 0.50), ASD (*p* = 0.99), ADHD (*p* = 0.23), MD (*p* = 0.99), AD (*p* = 0.99), and ED (*p* = 0.99) groups.

### 3.3. Language Disorder

In all SDQ Subscales, the majority of the participants with LD showed normal scores.

A comparison between groups revealed that participants with LD exhibited significantly better scores in the Emotional Problems Subscale than the group with ID (*p* < 0.0001), SLD (*p* < 0.0001), ASD (*p* < 0.01), ADHD (*p* < 0.0001), MD (*p* < 0.0001), AD (*p* < 0.0001), and ED (*p* < 0.0001) but did not differ from the group with GDD (*p* = 0.40).

In the Conduct Problems Subscale, they obtained significantly better scores than the group with only ADHD (*p* < 0.0001) but did not differ from the GDD (*p* = 0.99), ID (*p* = 0.21), SLD (*p* = 0.99), ASD (*p* = 0.99), MD (*p* = 0.05), AD (*p* = 0.99), and ED (*p* = 0.99) groups.

Similarly, they showed significantly better scores in the Hyperactivity/Inattention Subscale than the group with GDD (*p* < 0.0001), ASD (*p* < 0.0001), and ADHD (*p* < 0.0001) but did not differ from the ID (*p* = 0.12), SLD (*p* = 0.99), MD (*p* = 0.21), AD (*p* = 0.99), and ED (*p* = 0.86) groups.

Further, they exhibited significantly better scores in the Peer Problems Subscale than the group with ID (*p* < 0.0001), ASD (*p* < 0.0001), and MD (*p* < 0.0001) but did not differ from the GDD (*p* = 0.03), SLD (*p* = 0.99), ADHD (*p* = 0.23), AD (*p* = 0.99), and ED (*p* = 0.99) groups.

In the Prosocial Behaviour Subscale, they obtained significantly better scores than the group with GDD (*p* < 0.0001) and ASD (*p* < 0.0001) but did not differ from the ID (*p* = 0.99), SLD (*p* = 0.17), ADHD (*p* = 0.99), MD (*p* = 0.99), AD (*p* = 0.99), and ED (*p* = 0.99) groups.

### 3.4. Specific Learning Disorder

With the exception of the Emotional Problems Subscale, participants with SLD obtained normal scores in the remaining SDQ subscales.

A comparison between groups revealed that the participants with SLD exhibited significantly better scores in the Emotional Problems Subscale than the group with MD (*p* < 0.0001), while obtaining significantly worse scores than the LD group (*p* < 0.0001). However, they did not differ from the GDD (*p* = 0.04), ID (*p* = 0.99), ASD (*p* = 0.99), ADHD (*p* = 0.99), AD (*p* = 0.05), and ED (*p* = 0.50) groups.

In the Conduct Problems Subscale, they displayed significantly better scores than the group with ADHD (*p* < 0.0001) but did not differ from the GDD (*p* = 0.99), ID (*p* = 0.50), LD (*p* = 0.99), ASD (*p* = 0.99), MD (*p* = 0.15), AD (*p* = 0.99), and ED (*p* = 0.99) groups.

Similarly, they obtained significantly better scores in the Hyperactivity/Inattention Subscale than the group with ASD (*p* < 0.001) and ADHD (*p* < 0.0001) but did not differ from the GDD (*p* = 0.03), ID (*p* = 0.99), LD (*p* = 0.99), MD (*p* = 0.99), AD (*p* = 0.99), and ED (*p* = 0.02) groups.

In the Peer Problems Subscale, they also showed significantly better scores than the group with GDD (*p* = 0.009), ID (*p* < 0.0001), ASD (*p* < 0.0001), and MD (*p* < 0.0001) but did not differ from the LD (*p* = 0.99), ADHD (*p* = 0.07), AD (*p* = 0.99), and ED (*p* = 0.99) groups.

Further, in the Prosocial Behaviour Subscale, they obtained significantly better scores than the group with GDD (*p* < 0.0001), ASD (*p* < 0.0001), and ADHD (*p* = 0.001) but did not differ from the ID (*p* = 0.04), LD (*p* = 0.17), MD (*p* = 0.99), AD (*p* = 0.99), and ED (*p* = 0.99) groups.

### 3.5. Autism Spectrum Disorder

The majority of participants with ASD showed clinical/borderline scores in the Hyperactivity/Inattention, Peer Problems, and Prosocial Behaviour Subscales. Especially in the Problems with Peers Subscale, the percentage of clinical scores was particularly substantial, reaching 69.6%.

A comparison between the groups revealed that participants with ASD exhibited significantly worse scores than the group with LD (*p* = 0.003) and better scores than the MD group (*p* < 0.0001) in the Emotional Problems Subscale, while they did not differ from the GDD (*p* = 0.99), ID (*p* = 0.99), SLD (*p* = 0.99), AD (*p* = 0.03), and ED (*p* = 0.16) groups.

In the Hyperactivity/Inattention Subscale, they displayed significantly worse scores than the groups with LD (*p* < 0.0001), AD (*p* < 0.0001), ED (*p* < 0.0001), and SLD (*p* < 0.001), but they did not differ from the GDD (*p* = 0.99), ID (*p* = 0.99), ADHD (*p* = 0.99), and MD (*p* = 0.99) groups.

In the Peer Problems Subscale, they also obtained significantly worse scores than the groups with GDD (*p* < 0.0001), LD (*p* < 0.0001), SLD (*p* < 0.0001), ADHD (*p* < 0.0001), AD (*p* < 0.0001), and ED (*p* < 0.001), but they did not differ from the MD group (*p* = 0.99).

Further, they showed significantly worse scores in the Prosocial Behaviour Subscale than the groups with ID (*p* = 0.006), LD (*p* < 0.0001), SLD (*p* < 0.0001), ADHD (*p* < 0.0001), MD (*p* < 0.0001), AD (*p* < 0.0001), and ED (*p* < 0.0001) but did not differ from the GDD group (*p* = 0.99).

Conversely, they obtained significantly better scores in the Conduct Problems Subscale than the group with ADHD (*p* < 0.0001) but did not differ from the GDD (*p* = 0.99), ID (*p* = 0.99), LD (*p* = 0.99), SLD (*p* = 0.99), MD (*p* = 0.99), AD (*p* = 0.99), and ED (*p* = 0.99) groups.

### 3.6. Attention Deficit/Hyperactivity Disorder

On the Conduct Problems and Hyperactivity/Inattention Subscales, more than two-thirds of the children and adolescents with ADHD showed borderline and clinical scores.

A comparison between the groups revealed that participants with ADHD exhibited significantly worse scores in the Emotional Problems Subscale than the group with LD (*p* < 0.0001) and significantly better scores than the group with MD (*p* < 0.0001). However, no other differences were found with the GDD (*p* = 0.48), ID (*p* = 0.99), SLD (*p* = 0.99), ASD (*p* = 0.99), AD (*p* = 0.02), and ED (*p* = 0.22) groups.

In the Conduct Problems Subscale, they displayed significantly worse scores than the groups with GDD (*p* < 0.0001), LD (*p* < 0.0001), SLD (*p* < 0.0001), ASD (*p* < 0.0001), AD (*p* < 0.0001), and ED (*p* = 0.003), but they did not differ from the ID (*p* = 0.23) and MD (*p* = 0.34) groups.

Similarly, in the Hyperactivity/Inattention Subscale, they obtained significantly worse scores than the groups with LD (*p* < 0.0001), SLD (*p* < 0.0001), AD (*p* < 0.0001), and ED (*p* < 0.0001) but did not differ from the GDD (*p* = 0.04), ID (*p* = 0.08), ASD (*p* = 0.99), and MD (*p* = 0.01) groups.

In the Prosocial Behaviour Subscale, the group with ADHD displayed significantly worse scores than the group with SLD (*p* = 0.001) and significantly better scores than the group with ASD (*p* < 0.0001). However, in the Prosocial Behaviour Subscale, they did not differ from the GDD (*p* = 0.01), ID (*p* = 0.99), LD (*p* = 0.99), MD (*p* = 0.99), AD (*p* = 0.22), and ED (*p* = 0.72) groups.

Conversely, they displayed significantly better scores in the Peer Problems Subscale than the group with ASD (*p* < 0.0001) but not compared to the GDD (*p* = 0.99), ID (*p* = 0.20), LD (*p* = 0.23), SLD (*p* = 0.07), MD (*p* = 0.32), AD (*p* = 0.99), and ED (*p* = 0.99) groups.

### 3.7. Mood Disorder

With the exception of the Hyperactivity/Inattention and Prosocial Behaviour Subscales, the majority of the participants with MD obtained clinical or borderline scores in the Emotional Problems, Conduct Problems, and Peer Problems Subscales. Of note, the percentage of clinical scores reached 74.5% on the Emotional Problems Subscale.

A comparison between the groups revealed that the participants with MD exhibited significantly worse scores in the Emotional Problems Subscale than the groups with GDD (*p* < 0.0001), LD (*p* < 0.0001), SLD (*p* < 0.0001), ASD (*p* < 0.0001), and ADHD (*p* < 0.0001) but did not differ from the groups with ID (*p* = 0.02), AD (*p* = 0.03), and ED (*p* = 0.99).

In the Hyperactivity/Inattention Subscale, they displayed significantly worse scores than the ED group (*p* = 0.004) but did not differ from the GDD (*p* = 0.99), ID (*p* = 0.99), LD (*p* = 0.99), SLD (*p* = 0.99), ASD (*p* = 0.99), ADHD (*p* = 0.01), and AD (*p* = 0.66) groups.

Further, in the Peer Problems Subscale, they showed significantly worse scores than the groups with LD (*p* < 0.0001) and SLD (*p* < 0.0001) but did not differ from the GDD (*p* = 0.99), ID (*p* = 0.99), ASD (*p* = 0.99), ADHD (*p* = 0.32), AD (*p* = 0.02), and ED (*p* = 0.68) groups.

Conversely, in the Prosocial Behaviour Subscale, they showed significantly better scores than the groups with GDD (*p* = 0.005) and ASD (*p* < 0.0001) but did not differ from the ID (*p* = 0.99), LD (*p* = 0.99), SLD (*p* = 0.99), ADHD (*p* = 0.99), AD (*p* = 0.99), and ED (*p* = 0.99) groups.

In the Conduct Problems Subscale, no differences were found for the MD group compared to the GDD (*p* = 0.99), ID (*p* = 0.99), LD (*p* = 0.05), SLD (*p* = 0.15), ASD (*p* = 0.99), ADHD (*p* = 0.34), AD (*p* = 0.99), and ED (*p* = 0.99) groups.

### 3.8. Anxiety Disorder

As expected, two-thirds of the participants with AD obtained clinical or borderline scores in the Emotional Problems Subscale. Approximately half of them also obtained clinical or borderline scores in the Conduct Problems Subscale.

A comparison between the groups revealed that participants with AD exhibited significantly worse scores only in the Emotional Problems Subscale than the groups with GDD (*p* < 0.0001) and LD (*p* < 0.0001), but they did not differ from the groups with ID (*p* = 0.99), SLD (*p* = 0.05), ASD (*p* = 0.03), ADHD (*p* = 0.02), MD (*p* = 0.03), and ED (*p* = 0.99).

Conversely, they showed significantly better scores in the Conduct Problems Subscale than the ADHD group (*p* < 0.0001) but did not differ from the GDD (*p* = 0.99), ID (*p* = 0.99), LD (*p* = 0.99), SLD (*p* = 0.99), ASD (*p* = 0.99), MD (*p* = 0.99), and ED (*p* = 0.99) groups.

Similarly, in the Hyperactivity Subscale, they obtained significantly better scores than the groups with GDD (*p* < 0.001), ASD (*p* < 0.0001), and ADHD (*p* < 0.0001), but no differences emerged compared to the groups with ID (*p* = 0.36), LD (*p* = 0.99), SLD (*p* = 0.99), MD (*p* = 0.66), and ED (*p* = 0.41).

In the Peer Problems Subscale, they also displayed significantly better scores than the group with ASD (*p* < 0.0001) but not compared to the GDD (*p* = 0.99), ID (*p* = 0.01), LD (*p* = 0.99), SLD (*p* = 0.99), ADHD (*p* = 0.99), MD (*p* = 0.02), and ED (*p* = 0.99) groups.

Further, they obtained significantly better scores in the Prosocial Behaviour Subscale than the groups with GDD (*p* < 0.0001) and ASD (*p* < 0.0001), but they did not differ from the ID (*p* = 0.80), LD (*p* = 0.99), SLD (*p* = 0.99), ADHD (*p* = 0.22), MD (*p* = 0.99), and ED (*p* = 0.99) groups.

### 3.9. Eating Disorders

Three-quarters of the participants with ED obtained clinical or borderline scores in the Emotional Problems Subscale, and more than half of them also obtained clinical or borderline scores in the Conduct Problems Subscale.

A comparison between the groups revealed that the participants with ED displayed significantly worse scores in the Emotional Problems Subscale than the groups with GDD (*p* < 0.001) and LD (*p* < 0.0001) but did not differ from the groups with ID (*p* = 0.99), SLD (*p* = 0.49), ASD (*p* = 0.16), ADHD (*p* = 0.22), MD (*p* = 0.99), and AD (*p* = 0.99).

Conversely, they obtained significantly better scores in the Conduct Problems Subscale than the group with only ADHD (*p* < 0.003), while they were not different from the GDD (*p* = 0.99), ID (*p* = 0.99), LD (*p* = 0.99), SLD (*p* = 0.99), ASD (*p* = 0.99), MD (*p* = 0.99), and AD (*p* = 0.99) groups.

In the Hyperactivity/Inattention Subscale, they also showed significantly better scores than the groups with GDD (*p* < 0.0001), ID (*p* = 0.002), ASD (*p* < 0.0001), ADHD (*p* < 0.0001), and MD (*p* < 0.01) but did not differ from the LD (*p* = 0.86), SLD (*p* = 0.02), and AD (*p* = 0.41) groups.

Similarly, they obtained significantly better scores in the Peer Problems Subscale than the group with ASD (*p* < 0.001) but did not differ from the GDD (*p* = 0.99), ID (*p* = 0.45), LD (*p* = 0.99), SLD (*p* = 0.99), ADHD (*p* = 0.99), MD (*p* = 0.68), and AD (*p* = 0.99) groups.

They also exhibited significantly better scores in the Prosocial Behaviour Subscale than the groups with GDD (*p* < 0.001) and ASD (*p* < 0.0001), but they did not differ from the ID (*p* = 0.90), LD (*p* = 0.99), SLD (*p* = 0.99), ADHD (*p* = 0.72), MD (*p* = 0.99), and AD (*p* = 0.99) groups.

### 3.10. Correlations between Age and SDQ Subscales

As shown in Table 2, age was significantly and positively correlated to the scores in the Emotional Problems Subscale in the groups with GDD (*p* < 0.0001), LD (*p* < 0.001), ASD (*p* < 0.001), ADHD (*p* < 0.001), and AD (*p* < 0.001), meaning that they displayed worse scores in the Emotional Problems Subscale as they grew older.

Age was significantly and negatively correlated to the scores in the Conduct Problems Subscale in the group with LD (*p* < 0.005), meaning that they showed better scores in the Conduct Problems Subscale as they grew older.

Moreover, age was significantly and positively correlated to the scores in the Peer Problems Subscale in the groups with ASD and AD (*p* always < 0.005), meaning that they displayed worse scores in the Peer Problems Subscale as they grew older.

Last, age was significantly and negatively correlated to the scores in the Prosocial Behaviour Subscale in the groups with GDD and LD (*p* always < 0.0001), meaning that they displayed better scores in the Prosocial Behaviour Subscale as they grew older.

No further correlations were found in the other diagnostic groups between age and scores on the SDQ subscales, as well as in the Hyperactivity/Inattention Subscale (*p* always > 0.005).

## 4. Discussion

The current study demonstrated that the SDQ is a valid tool for identifying relevant symptoms in our children and adolescents in accordance with neurodevelopmental and neuropsychiatric diagnosis, as well as their changes with age.

Firstly, the SDQ has the potential to detect the core symptoms of both ASD and ADHD, as it includes subscales that detect the core symptoms of ASD (e.g., peer and prosocial behaviour problems), as well as the core symptoms of ADHD (e.g., hyperactivity/attention problems). Indeed, we found that children and adolescents with ASD generally scored significantly worse than other groups on the subscales assessing difficulties in communication and social cognition, such as the Peer Problems and Prosocial Behaviour Subscales, and that, in the same subscales, the majority of them exhibited at-risk or clinically relevant scores. Our results are in line with previous findings [31] that showed the sensitivity of the Peer Problems Subscale in identifying the core symptoms of ASD, a subscale in which children with ASD generally tend to score worse than other groups.

It has been widely recognised that children with ASD have severe problems with peers, as supported by studies documenting fewer friends at school, less reciprocity of friendship, and narrower social networks than their classmates [46,47]. The vast majority of the studies reported that youths with ASD are less liked by peers and more likely to be rejected, ignored, and purposely excluded by their peer group. We also found that problems with peers have a significant and positive association with age, meaning that such problems seem to increase with age. Accordingly, the evidence indicated that behavioural problems are usually augmented during development and that, even, the majority of adults with ASD present with significant, ongoing social impairments [48]. Of note, more than half of the participants with ASD also had borderline or clinical scores on the Hyperactivity/Inattention subscale, indicating that the SDQ is sensitive to detecting symptoms that are highly associated with ASD but, before the DSM-5, could not be diagnosed in a comorbidity. Indeed, while DSM-IV-TR [37] gave mutually exclusive diagnoses of ADHD and ASD, DSM-5 allows a dual diagnosis of ASD and ADHD behaviours, admitting the possibility of a comorbidity. Accordingly, the literature described that ASD co-occurs with ADHD in 40–70% of cases [49,50], especially in pre-schoolers. Moreover, we found that difficulties in emotional symptoms have a significant and positive association with age, meaning that these symptoms seem to be augmented along with age. Our findings on correlations seem to agree with a wide range of studies that support the high rates of psychiatric disorders in ASD, such as depression and anxiety, occurring more in adulthood [51].

Considering ADHD, we found that children and adolescents with ADHD generally scored significantly worse than other groups on the subscales assessing hyperactive behaviour/inattention and conduct problems and that, in the same subscales, more than two-thirds of them exhibited at-risk or clinically relevant scores. Our results provided evidence that the Hyperactivity/Inattention Subscale of the SDQ showed good agreement with the diagnostic criteria for ADHD, in agreement with previous studies [30,31]. Consistent with our findings on conduct problems, many studies have documented that ADHD and oppositional defiant disorder or conduct disorder often cooccur in about 40–60% of cases [52]. Previous studies examining the covariations between ADHD, oppositional defiant disorder, and conduct disorder emphasised that a common genetic risk factor explains more than half of the variance among the disorders [52,53].

The SDQ also detected symptoms that are highly associated with ADHD, such as well-documented dysfunctional social abilities. Accordingly, we found that more than half of children and adolescents with ADHD obtained at-risk or clinically relevant scores on the Peer Problems Subscale. According to previous studies [31], this subscale of the SDQ allows us to highlight an aspect that is often associated with ADHD and is one of the main factors responsible for the impaired quality of life of individuals with ADHD. It has been widely reported that one of the main problems of children with ADHD are difficulties in establishing mutual friendships, peer rejection, and bullying—mainly due to impulsivity and a poor attention span, leading to serious problems in developing satisfactory social relationships with peers [54,55].

In addition, we found that the emotional symptoms increase with age, as documented by our results on correlations. Several studies have demonstrated a high likelihood of finding the psychopathological conditions associated with ADHD, including emotional problems such as anxiety and major depression [56,57]. These conditions are documented especially in adolescence and adulthood [56]. Our findings on correlations are in line with these studies in ADHD documenting the emotional symptoms during childhood and adolescence that worsen and are accompanied by an increased risk of depression in adulthood [56].

Considering neuropsychiatric disorders, we found that the SDQ has the potential to also detect the core symptoms of both MD and AD. Indeed, the majority of our children and adolescents with MD and AD obtained at-risk or clinically relevant scores and scored significantly worse than other groups on the Emotional Problems Subscale of the SDQ. This subscale measures negative physical or cognitive symptoms (i.e., headache and worries) and mood state (i.e., sadness, tension for new situations, fears, and easily scared), in accordance with the MD and AD diagnostic DSM-5 criteria [38]. Moreover, we observed that more than half of the participants with MD and AD exhibited at-risk or clinically relevant scores on the Conduct Problems Subscale, which assesses for disrupted behaviours, irritability, and emotional dysregulation symptoms, such as difficulties in managing anger and mood. In line with previous studies [58], we found that participants with MD and AD showed a similar profile in this subscale, obtaining worse scores than the other diagnostic groups. Besides the core symptoms of MD, we found that a relevant percentage of the children and adolescents with MD obtained at-risk or clinically relevant scores on the Peer Problems Subscale. It has been shown that children and adolescents with high levels of emotional problems, such as MD patients, have difficulty establishing meaningful and special relationships with peers [59].

Of note, correlations revealed that emotional and peer problems have significant and positive associations with age in participants with AD. This finding is in line with studies documenting that AD symptoms are greater with increasing age [60] and are accompanied by a change in social skills and motivation to socialise, which affects the creation, maintenance, and termination of social ties [61].

Considering children and adolescents with ED, we found that three-quarters of them had clinical scores on the Emotional Problems Subscale in agreement with studies reporting severe depressive symptoms, especially during adolescence in this population [62,63].

Regarding the other diagnostic groups for which the core symptoms could not be captured directly by the SDQ subscales, the tool was able to identify the transdiagnostic dimensions of neurodevelopmental and neuropsychiatric disorders, as well as changes in symptomatology with age.

Considering neurodevelopmental disorders, we found that scores on emotional problems in LD have a significant and positive association with age. Our results were in line with studies indicating that one area of particular vulnerability for children and adolescents with LD is emotional difficulties [64,65,66] that increase in symptomatology with age [67].

In addition, we found that participants with GDD and ID showed a rather similar profile on the Conduct Problems, Hyperactivity/Inattention, and Peer Problems Subscales. From a developmental perspective, we must consider that children presenting with GDD often result in ID, as is evident from the overlap in their symptomatology. The GDD and ID did not differ from the other neurodevelopmental disorders, such as ADHD and ASD, in the Hyperactivity/Inattention Subscale. Studies documented that children and adolescents with developmental disabilities were at a heightened risk for developing ADHD [68] and conduct problems [69]. In fact, behaviour disorders are common in children with cognitive delays, are often chronically disabling and can create problems in daily life. The SDQ has allowed us to also highlight these behavioural problems associated with disability and to guide parents toward the appropriate management and treatment of them. Similar to the other neurodevelopmental disorders we have discussed (i.e., ASD and ADHD), in groups with ID and GDD, developmental disabilities led to impaired social and relationship aspects, as documented by the worse scores on the Peer Problems Subscale. It should be noted that relational problems in GDD improve with growth, perhaps because the relational aspects are considered in therapeutic interventions [70]. In fact, for children whose cognitive abilities are impaired, the degree of relational problems can often be the difference between dependency and self-sufficiency, and interventions aimed at decreasing an individual’s vulnerability through social skill development are commonly critical components of any therapeutic plan. Differently from GDD, participants with ID did not differ from psychopathological disorders, such as MD, AD, and ED, in the Emotional Problems Subscale. This result was in line with previous evidence showing in individuals with ID levels of emotional symptoms approximately three to four times higher than those of typically developing children [71]. Indeed, children with ID are at greater risk for MD and AD, especially AD, one of the most common forms of social distress found in ID [72,73].

Regarding SLD, more than half of the participants with SLD showed borderline or clinical scores in the Emotional Problems Subscales. The presence of internalising symptoms (i.e., depression and anxiety) in SLD have been well-documented in the literature [74,75,76]. Emotional problems in SLD are usually a consequence of many years of academic frustrations and negative experiences during the school years, especially when appropriate accommodations, support services, or individualised and specialised teachings are not provided. The SDQ was able to capture these emotional symptoms in children and adolescents with SLD and adequately handle these associated problems as well.

Our study has some limitations.

A first limitation was the lack of a control group, which should be included in future studies to compare the prevalence of the symptoms we found with that of the typically developing population.

Another limitation was the absence of a complete description of our patients’ characteristics. Future studies should include a detailed description, including the demographic (e.g., socioeconomic status) and clinical characteristics of the patients (e.g., whether they are taking medication or not).

An additional limitation was the wide age range of participants. Including participants with a more homogeneous age range or stratifying them by age could better clarify the relationship between age and symptom development.

In addition, the number of participants considering each diagnostic group was limited, but the results are encouraging and useful for designing and executing a large-scale study using the SDQ.

Finally, future studies should include multi-informant analysis, considering self-reporting and teacher report questionnaires. It should augment the generalisability of the results into the daily professional’s routine.

Overall, our results highlighted the clinical utility of using the SDQ as a potential tool to conduct the initial screening, and then further refer to patients for more structured neuropsychiatric evaluation. This is especially true for the early identification of core symptoms of ASD, ADHD, MD, and AD. Clinical assessments, such as the neuropsychiatric assessment, are complex and expensive. The SDQ can be integrated into daily clinical practice as an initial filter and then refer for the structured assessment only patients who score at-risk or clinical on the SDQ and who need further investigation.

Our results also showed that SDQ can detect behavioural and emotional problems that may be associated, with GDD, ID, SLD, and ED. Without a doubt, research on comorbid neuropsychiatric disorders has advanced in recent decades. However, because the comorbidity is associated with the cognitive and behavioural deficits of the primary disorder and influences its development; treatment should also focus on the comorbidities. The SDQ can bring precisely more attention to the associated symptoms to develop intervention plans that also address the comorbid problems.

## 5. Conclusions

In summary, our results indicated that the SDQ is a valid parent report questionnaire for identifying diagnostic and associated symptoms in the children and adolescents assessed in our study.

Specifically, the SDQ allowed us to detect specific symptoms for ascertaining the diagnosis in children and adolescents with ADHD, ASD, MD, and AD. In addition, SDQ can detect transdiagnostic emotional and behavioural problems associated with neurodevelopmental and neuropsychiatric disorders to better guide parents in their management.

## Figures and Tables

**Table 1:** Prevalence (%) and number of children and adolescents who showed normal, borderline, and clinical scores in the SDQ subscales for each diagnostic group, as well as the mean and standard deviation (SD) of the scores in the SDQ subscales and Post hoc comparisons.

**Diagnostic Groups**	**Emotional Problems** normal (0–3) borderline (4) clinical (5–10)	**Conduct Problems** normal (0–2) borderline (3) clinical (4–10)	**Hyperactivity/Inattention** normal (0–5) borderline (6) clinical (7–10)	**Peer Problems** normal (0–2) borderline (3) clinical (4–10)	**Prosocial Behaviour** normal (0–4) borderline (5) clinical (6–10)
	M (SD) Post hoc comparisons	M (SD) Post hoc comparisons	M (SD) Post hoc comparisons	M (SD) Post hoc comparisons	M (SD) Post hoc comparisons
Global Developmental Delay	N = 64.6% (73) B = 13.3% (15) C = 22.1% (25)	N = 50.4% (57) B = 17.7% (20) C = 31.9% (36)	N = 48.7% (55) B = 13.3% (15) C = 38% (43)	N = 40.7% (46) B = 17.7% (20) C = 41.6% (47)	N = 56.6% (64) B = 11.5% (13) C = 31.9% (36)
	2.8 (2.15)	2.9 (2.17)	5.5 (2.76)	3.1 (2.06)	4.4 (2.66)
	<MD *** <AD *** <ED **	<ADHD ***	>LD *** >AD ** >ED ***	>SLD * <ASD ***	>LD *** >SLD *** >MD * >AD *** >ED **
Intellectual Disability	N = 44.4% (20)B = 6.7% (3) C = 48.9% (22)	N = 40% (18)B = 11.1% (5) C = 48.9% (22)	N = 53.3% (24)B = 11.1% (5) C = 35.6% (16)	N = 24.4% (11)B = 15.6% (7) C = 60% (27)	N = 66.7% (30)B = 6.6% (3) C = 26.7% (12)
	4.3 (2.80)	3.4 (2.44)	5.2 (3.03)	4.2 (2.56)	3.6 (3.01)
	>LD ***		>ED *	>LD *** >SLD ***	<ASD *
Language Disorder	N = 78.5% (135) B = 11% (19) C = 10.5% (18)	N = 57.6% (99) B = 18% (31) C = 24.4% (42)	N = 75.6% (130) B = 9.9% (17) C = 14.5% (25)	N = 58.7% (101) B = 17.4% (30) C = 23.9% (41)	N = 80.2% (138) B = 9.3% (16) C = 10.5% (18)
	2.0 (1.87)	2.4 (1.89)	3.8 (2.50)	2.2 (1.88)	2.7 (2.23)
	<ID *** <SLD *** <ASD * <ADHD *** <MD *** <AD *** <ED ***	<ADHD ***	<GDD *** <ASD *** <ADHD ***	<ID *** <ASD *** <MD ***	<GDD *** <ASD ***
Specific Learning Disorder	N = 46% (86) B = 15.5% (29) C = 38.5% (72)	N = 56.2% (105) B = 16.5% (31) C = 27.3% (51)	N = 67.4% (126) B = 10.7% (20) C = 21.9% (41)	N = 64.7% (121) B = 13.9% (26) C = 21.4% (40)	N = 88.2% (165) B = 4.8% (9) C = 7% (13)
	3.7 (2.41)	2.5 (2.03)	4.4 (2.70)	2.2 (2.14)	2.1 (2.10)
	>LD *** <MD ***	<ADHD ***	<ASD ** <ADHD ***	<GDD * <ID *** <ASD *** <MD ***	<GDD *** <ASD *** <ADHD ***
Autism Spectrum Disorder	N = 53.6% (37) B = 18.8% (13) C = 27.6% (19)	N = 55.1% (38) B = 15.9% (11) C = 29% (20)	N = 34.8% (24) B = 24.6% (17) C = 40.6% (28)	N = 11.6% (8) B = 18.8% (13) C = 69.6% (48)	N = 39.1% (27) B = 11.6% (8) C = 49.3% (34)
	3.4 (2.47)	2.7 (1.93)	6.1 (1.94)	4.9 (2.06)	5.3 (2.46)
	>LD * <MD ***	<ADHD ***	>LD *** >AD *** >ED *** >SLD **	>GDD *** >LD *** >SLD *** >ADHD *** >AD *** >ED **	>ID * >LD *** >SLD *** >ADHD *** >MD *** >AD *** >ED ***
Attention Deficit/ Hyperactivity Disorder	N = 51.9% (60) B = 9.9% (13) C = 38.2% (50)	N = 19.8% (26) B = 13% (17) C = 67.2% (88)	N = 29% (38) B = 15.3% (20) C = 55.7% (73)	N = 47.3% (62) B = 13.7% (18) C = 39% (51)	N = 70.2% (92) B = 14.5% (19) C = 15.3% (20)
	3.6 (2.62)	4.5 (2.19)	6.7 (2.44)	2.9 (2.18)	3.1 (2.37)
	>LD *** <MD ***	>GDD *** >LD *** >SLD *** >ASD *** >AD *** >ED **	>LD *** >SLD *** >AD *** >ED ***	<ASD ***	>SLD ** <ASD ***
Mood Disorder	N = 13.7% (7) B = 11.8% (6) C = 74.5% (38)	N = 31.4% (16) B = 27.4% (14) C = 41.2% (21)	N = 62.7% (32) B = 9.8% (5) C = 27.5% (14)	N = 27.5% (14) B = 19.6% (10) C = 52.9% (27)	N = 82.4% (42) B = 7.8% (4) C = 9.8% (5)
	6.5 (2.50)	3.5 (2.10)	5.1 (2.70)	4.0 (2.40)	2.6 (1.90)
	>GDD *** >LD ***>SLD *** >ASD *** >ADHD ***		>ED *	>LD *** >SLD ***	<GDD * <ASD ***
Anxiety Disorder	N = 33.8% (51) B = 11.2% (17) C = 55% (83)	N = 48.3% (73) B = 19.2% (29) C = 32.5% (49)	N = 72.8% (110) B = 9.3% (14) C = 17.9% (27)	N = 51.6% (78) B = 13.3% (20) C = 35.1% (53)	N = 83.4% (126) B = 6.6% (10) C = 10% (15)
	4.8 (2.83)	2.9 (2.11)	4.0 (2.64)	2.7 (2.26)	2.4 (2.18)
	>GDD *** >LD ***	<ADHD ***	<GDD ** <ASD *** <ADHD ***	<ASD ***	<GDD *** <ASD ***
Eating Disorders	N = 27.3% (9) B = 18.2% (6) C = 54.5% (18)	N = 45.5% (15) B = 24.2% (8) C = 30.3% (10)	N = 87.9% (29) B = 3% (1) C = 9.1% (3)	N = 51.5% (17) B = 15.2% (5) C = 33.3% (11)	N = 90.9% (30) B = 3% (1) C = 6.1% (2)
	5.1 (2.46)	2.8 (2.37)	2.6 (2.60)	2.8 (2.24)	2.0 (1.83)
	>GDD ** >LD ***	<ADHD *	<GDD *** <ID ** <ASD *** <ADHD *** <MD *	<ASD **	<GDD ** <ASD ***

After Bonferroni’s correction: * *p*-value < 0.01; ** *p*-value < 0.001; *** *p*-value < 0.0001. Only significant differences are reported. N: normal; B: borderline; C: clinical; <: better scores than those of other groups; >: worse scores than those of other groups.

**Table 2:** Correlations (Spearman’s rho) between age and scores on the SDQ subscales for each diagnostic group.

		Emotional Problems *rho*	Conduct Problems *rho*	Hyperactivity/ Inattention *rho*	Peer Problems *rho*	Prosocial Behaviour *rho*
**Age**	Global Developmental Delay	0.45 ***	0.07	0.13	0.11	−0.40 ***
	Intellectual Disability	0.033	0.04	−0.19	0.30	0.06
	Language Disorders	0.25 **	−0.23 *	−0.03	−0.14	−0.41 ***
	Specific Learning Disorders	0.06	0.05	−0.04	0.07	0.02
	Autism Spectrum Disorder	0.43 **	0.13	0.22	0.35 *	−0.14
	Attention Deficit/Hyperactivity Disorder	0.33 ***	0.10	0.22	0.06	0.05
	Mood Disorder	0.13	0.12	−0.10	0.19	−0.07
	Anxiety Disorders	0.40 ***	0.05	−0.02	0.26 *	−0.18
	Eating Disorders	0.20	0.18	−0.08	−0.06	0.06

After Bonferroni’s correction: * *p*-value < 0.005; ** *p*-value < 0.001; *** *p*-value < 0.0001.

## Data Availability

The data presented in this study are available on request from the corresponding author.
